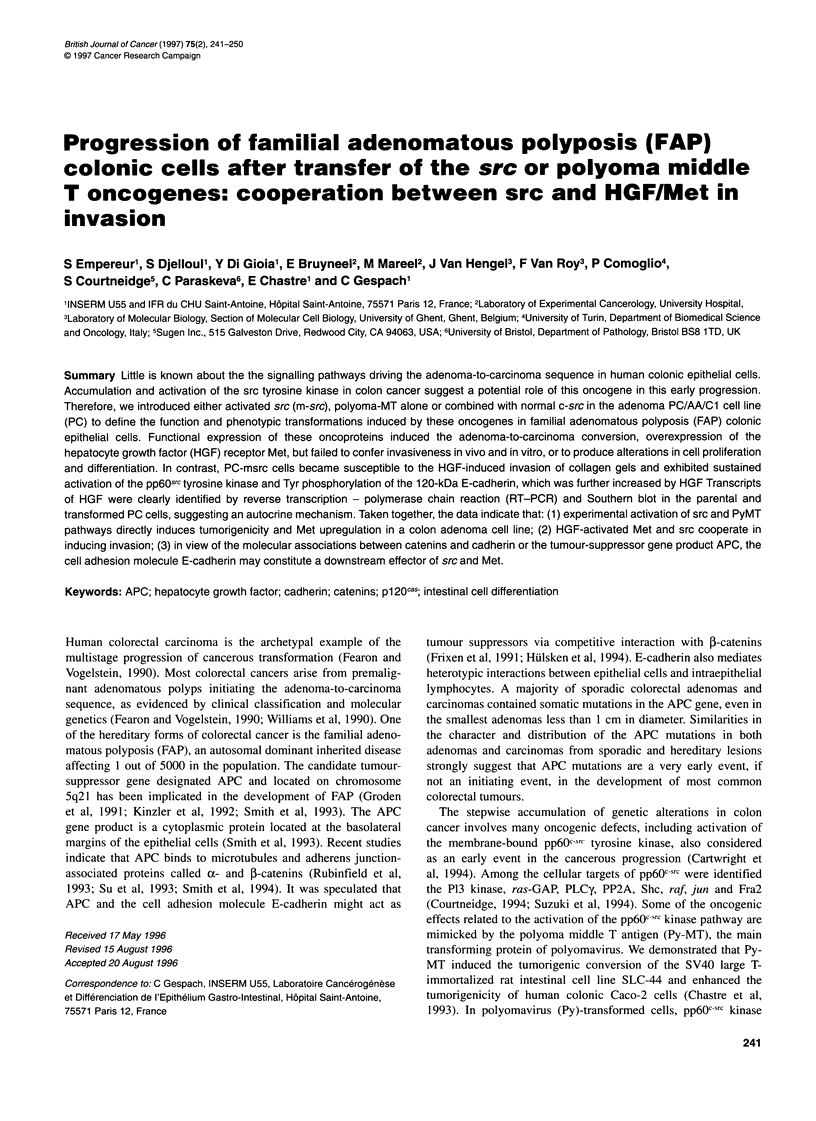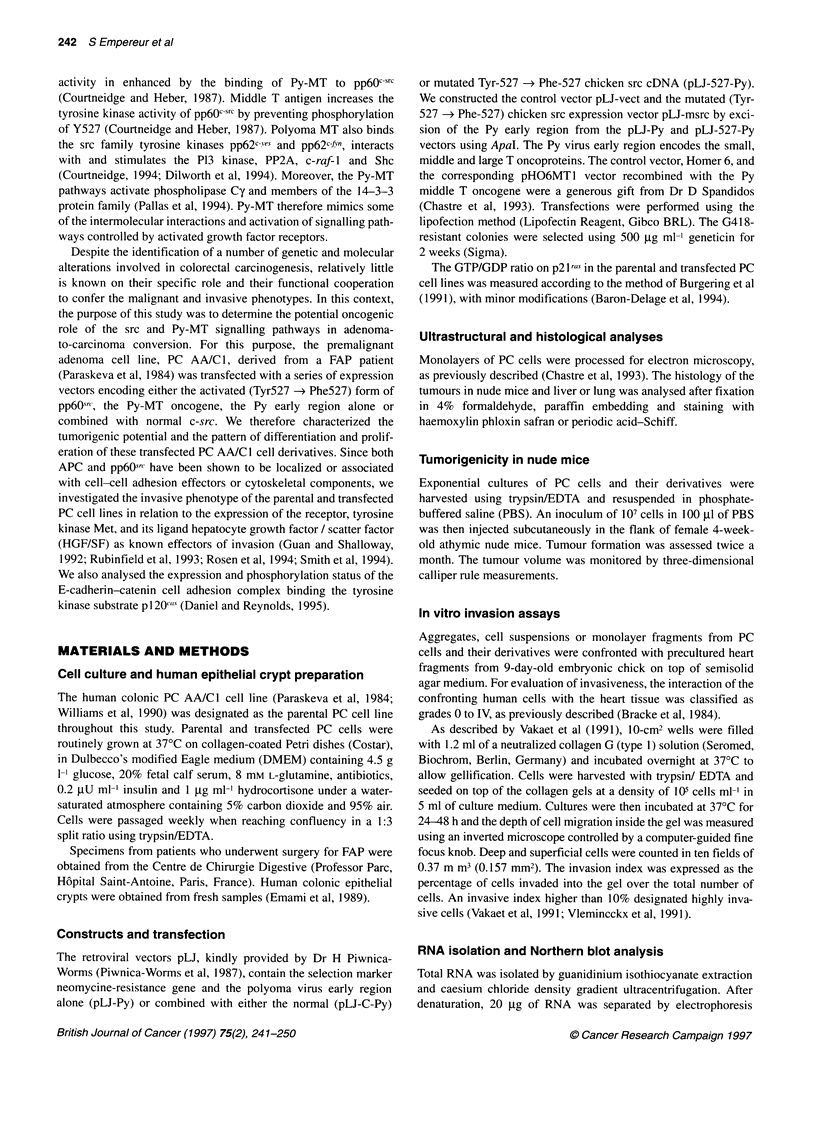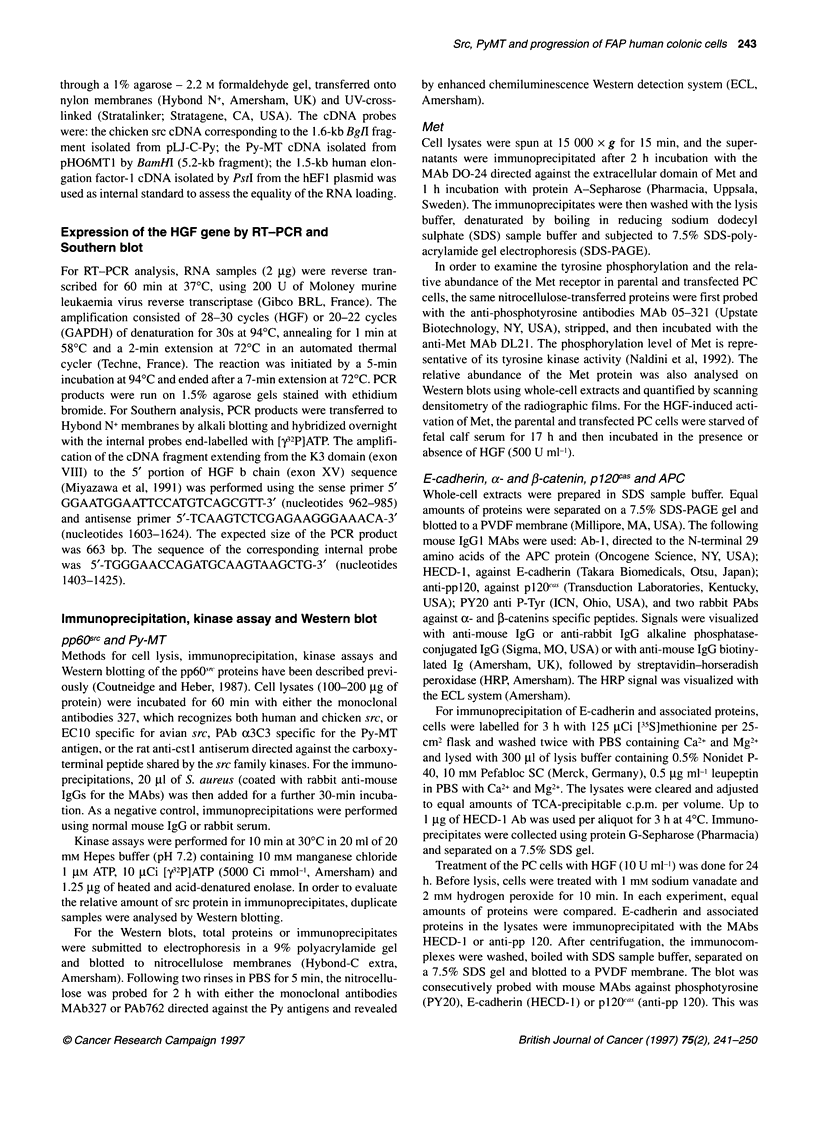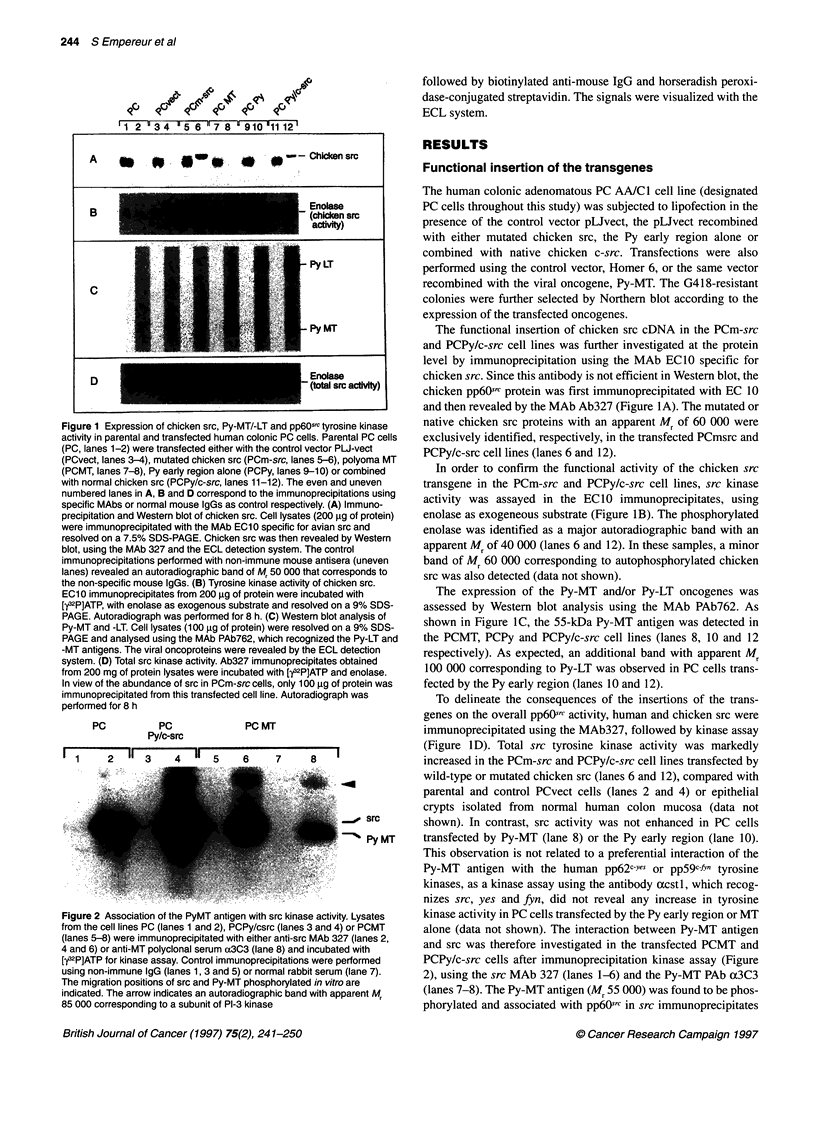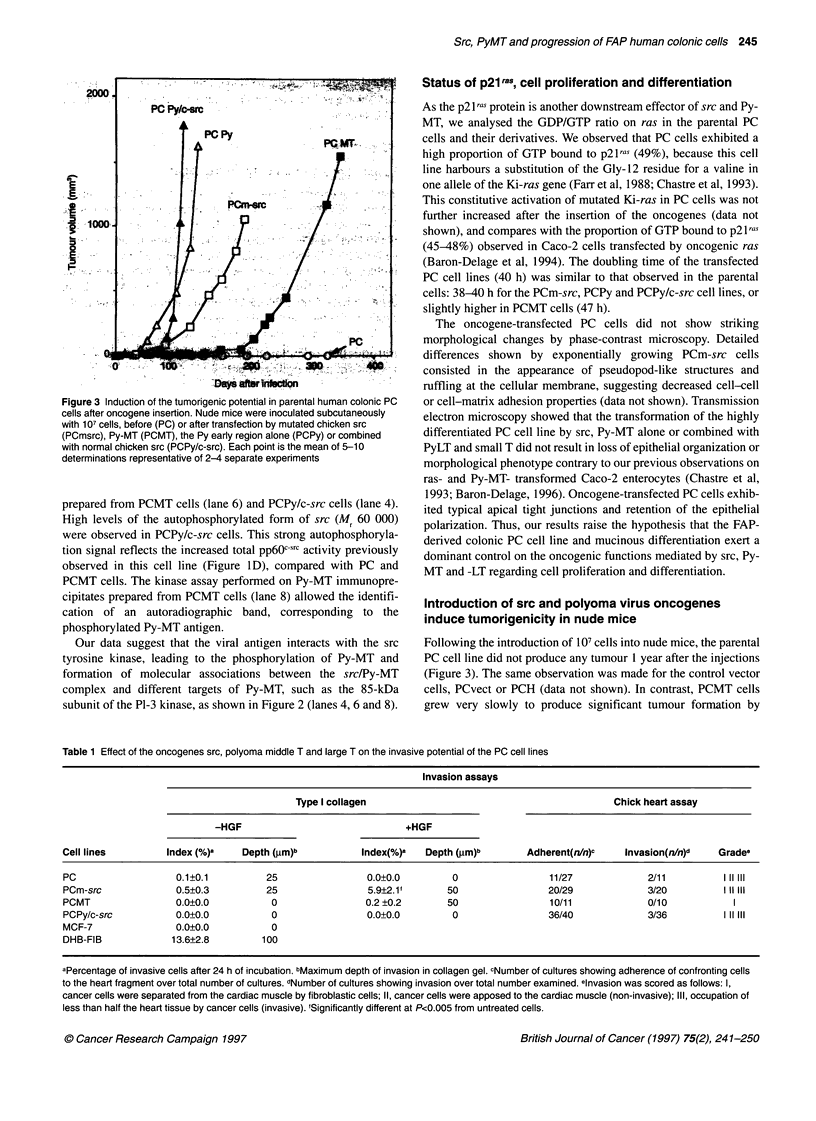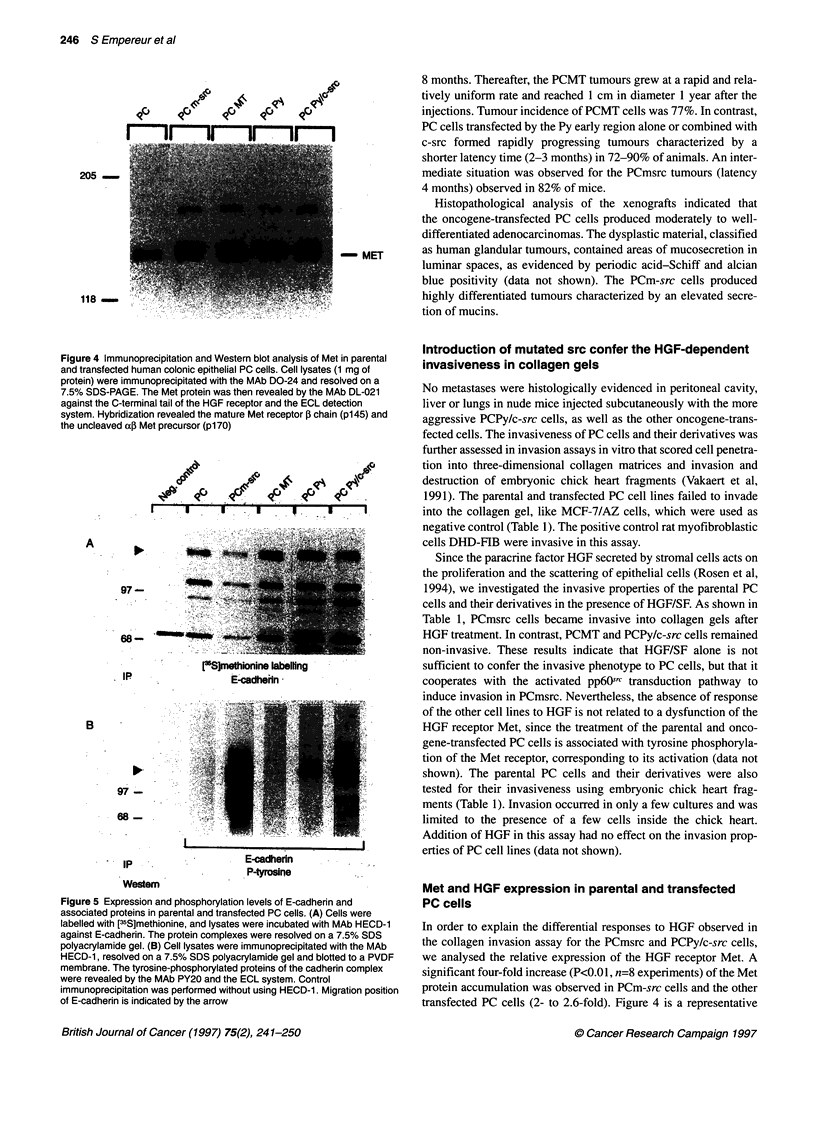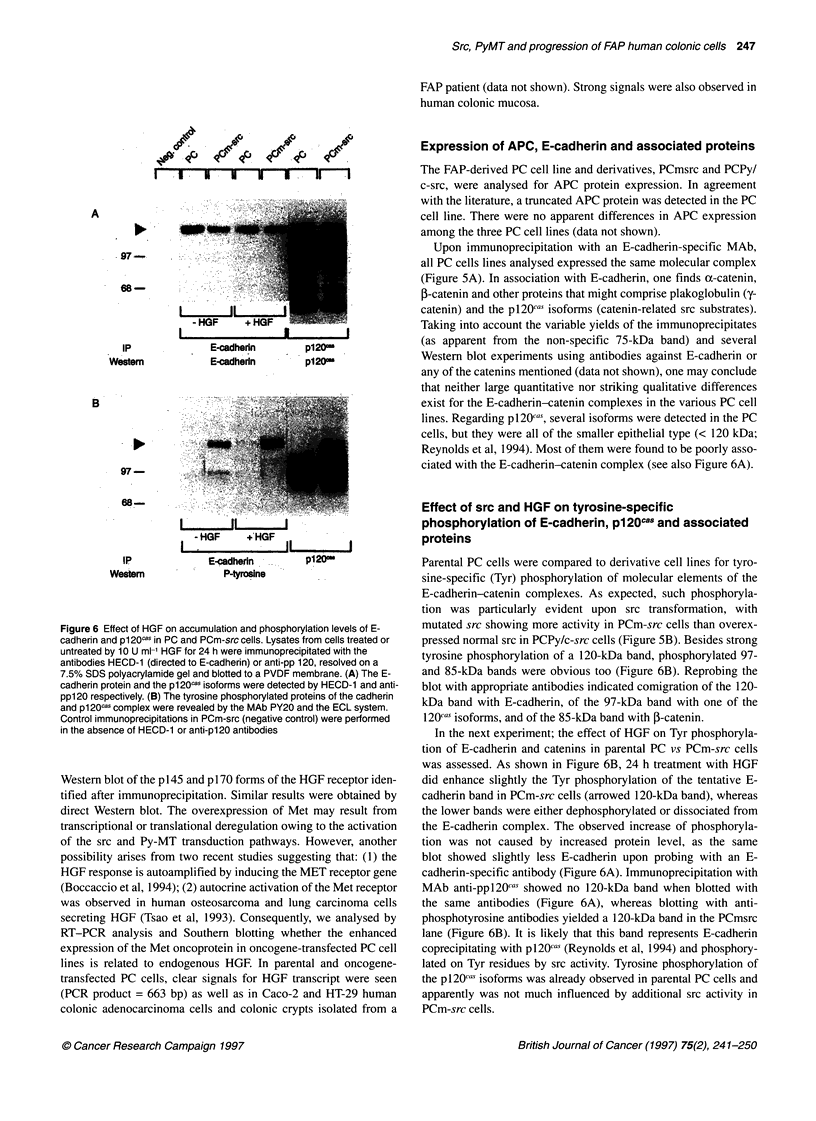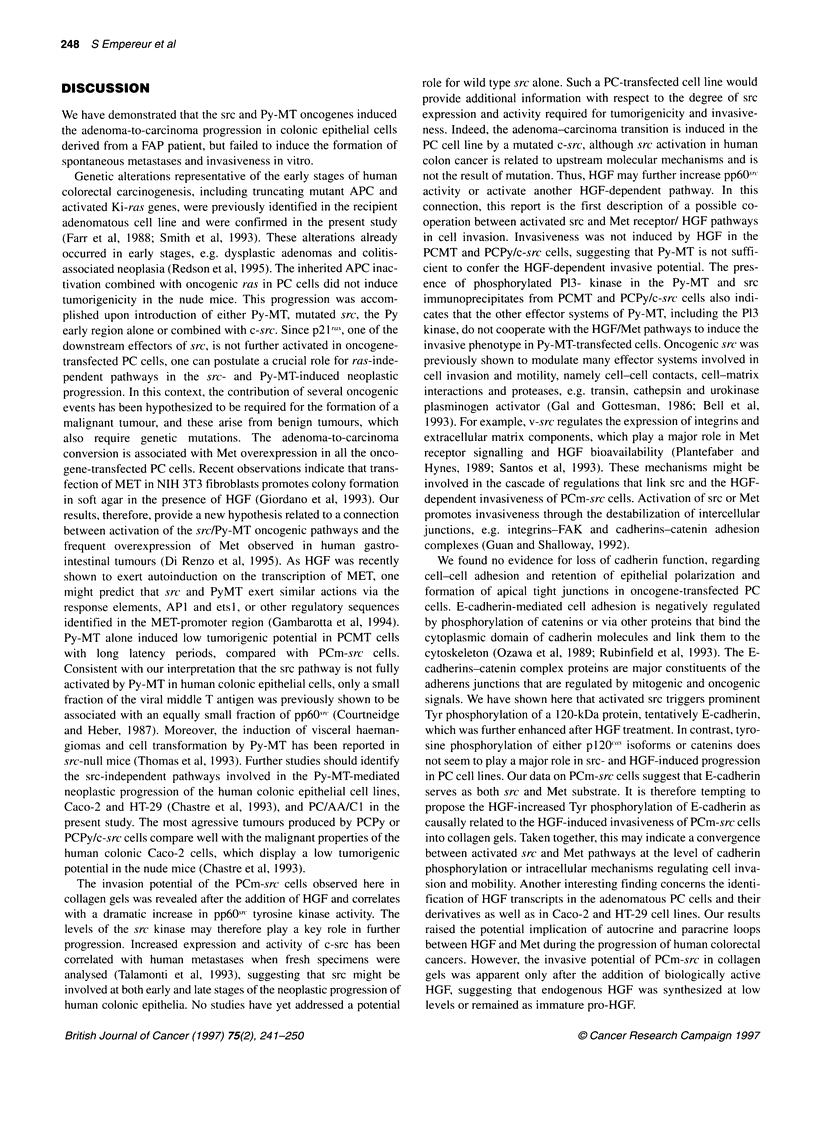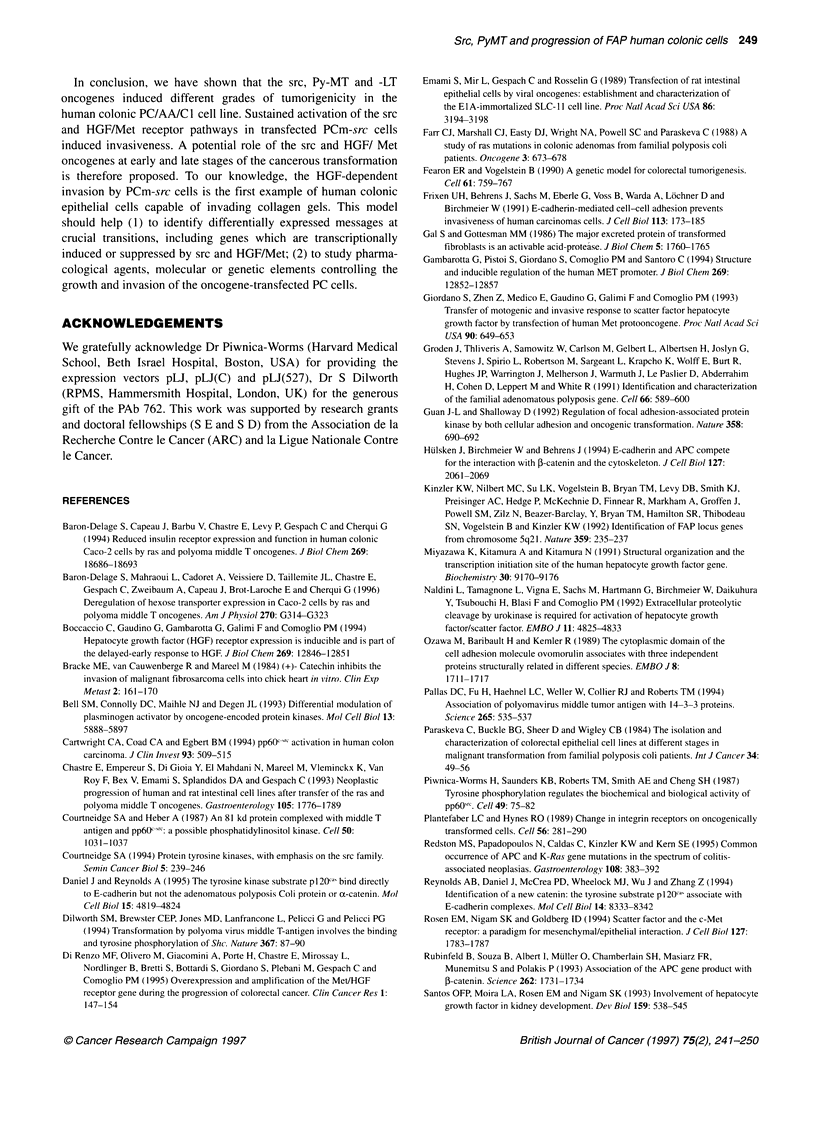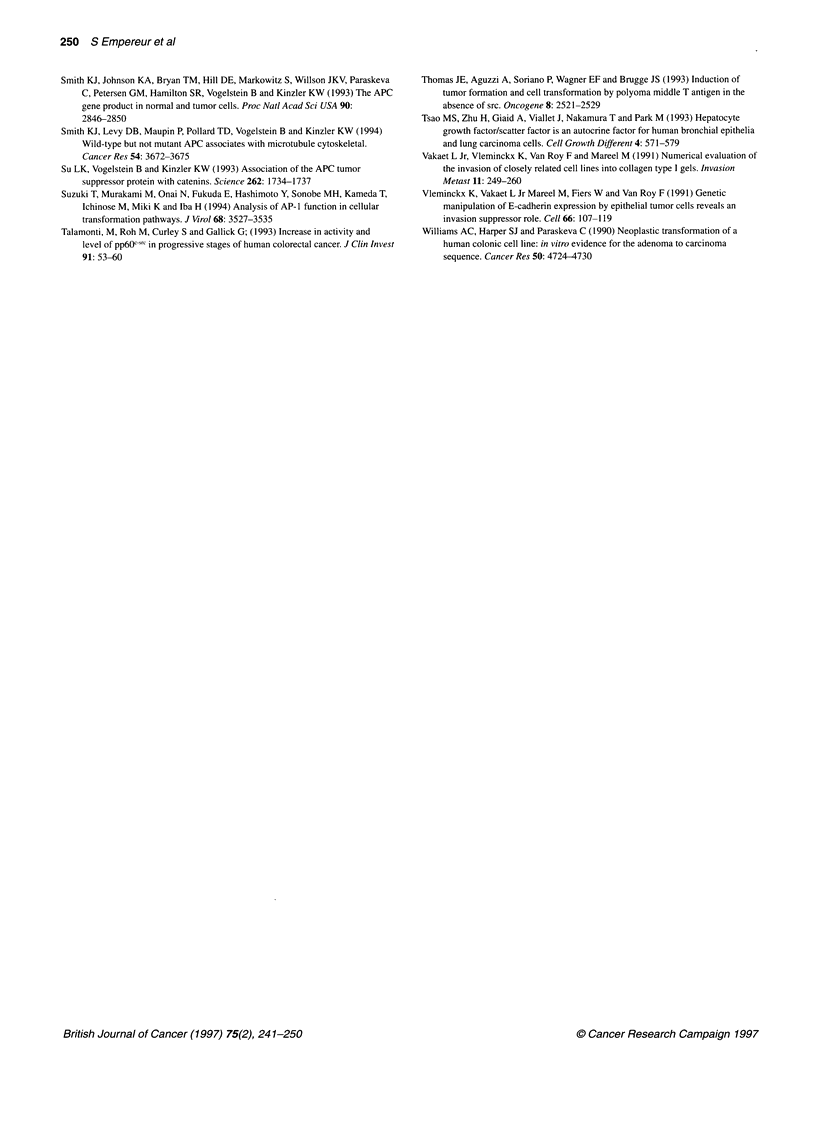# Progression of familial adenomatous polyposis (FAP) colonic cells after transfer of the src or polyoma middle T oncogenes: cooperation between src and HGF/Met in invasion.

**DOI:** 10.1038/bjc.1997.40

**Published:** 1997

**Authors:** S. Empereur, S. Djelloul, Y. Di Gioia, E. Bruyneel, M. Mareel, J. Van Hengel, F. Van Roy, P. Comoglio, S. Courtneidge, C. Paraskeva, E. Chastre, C. Gespach

**Affiliations:** INSERM U55, Hôpital Saint-Antoine, Paris, France.

## Abstract

**Images:**


British Journal of Cancer (1997) 75(2), 241–250
© 1997 Cancer Research Campaign

### Progression of familial adenomatous polyposis (FAP) colonic cells after transfer of the *src* or polyoma middle T oncogenes: cooperation between src and HGF/Met in invasion

S Empereur[1], S Djelloul[1], Y Di Gioia[1], E Bruyneel[2], M Mareel[2], J Van Hengel[3], F Van Roy[3], P Comoglio[4], S Courtneidge[5], C Paraskeva[6], E Chastre[1] and C Gespach[1]

[1]INSERM U55 and IFR du CHU Saint-Antoine, Hôpital Saint-Antoine, 75571 Paris 12, France; [2]Laboratory of Experimental Cancerology, University Hospital, [3]Laboratory of Molecular Biology, Section of Molecular Cell Biology, University of Ghent, Ghent, Belgium; [4]University of Turin, Department of Biomedical Science and Oncology, Italy; [5]Sugen Inc., 515 Galveston Drive, Redwood City, CA 94063, USA; [6]University of Bristol, Department of Pathology, Bristol BS8 1TD, UK

**Summary** Little is known about the the signalling pathways driving the adenoma-to-carcinoma sequence in human colonic epithelial cells. Accumulation and activation of the src tyrosine kinase in colon cancer suggest a potential role of this oncogene in this early progression. Therefore, we introduced either activated *src* (m-*src*), polyoma-MT alone or combined with normal c-*src* in the adenoma PC/AA/C1 cell line (PC) to define the function and phenotypic transformations induced by these oncogenes in familial adenomatous polyposis (FAP) colonic epithelial cells. Functional expression of these oncoproteins induced the adenoma-to-carcinoma conversion, overexpression of the hepatocyte growth factor (HGF) receptor Met, but failed to confer invasiveness in vivo and in vitro, or to produce alterations in cell proliferation and differentiation. In contrast, PC-msrc cells became susceptible to the HGF-induced invasion of collagen gels and exhibited sustained activation of the $pp60^{src}$ tyrosine kinase and Tyr phosphorylation of the 120-kDa E-cadherin, which was further increased by HGF Transcripts of HGF were clearly identified by reverse transcription – polymerase chain reaction (RT–PCR) and Southern blot in the parental and transformed PC cells, suggesting an autocrine mechanism. Taken together, the data indicate that: (1) experimental activation of src and PyMT pathways directly induces tumorigenicity and Met upregulation in a colon adenoma cell line; (2) HGF-activated Met and src cooperate in inducing invasion; (3) in view of the molecular associations between catenins and cadherin or the tumour-suppressor gene product APC, the cell adhesion molecule E-cadherin may constitute a downstream effector of *src* and Met.

**Keywords:** APC; hepatocyte growth factor; cadherin; catenins; $p120^{cas}$; intestinal cell differentiation

Human colorectal carcinoma is the archetypal example of the multistage progression of cancerous transformation (Fearon and Vogelstein, 1990). Most colorectal cancers arise from premalignant adenomatous polyps initiating the adenoma-to-carcinoma sequence, as evidenced by clinical classification and molecular genetics (Fearon and Vogelstein, 1990; Williams et al, 1990). One of the hereditary forms of colorectal cancer is the familial adenomatous polyposis (FAP), an autosomal dominant inherited disease affecting 1 out of 5000 in the population. The candidate tumour-suppressor gene designated APC and located on chromosome 5q21 has been implicated in the development of FAP (Groden et al, 1991; Kinzler et al, 1992; Smith et al, 1993). The APC gene product is a cytoplasmic protein located at the basolateral margins of the epithelial cells (Smith et al, 1993). Recent studies indicate that APC binds to microtubules and adherens junction-associated proteins called α- and β-catenins (Rubinfield et al, 1993; Su et al, 1993; Smith et al, 1994). It was speculated that APC and the cell adhesion molecule E-cadherin might act as tumour suppressors via competitive interaction with β-catenins (Frixen et al, 1991; Hülsken et al, 1994). E-cadherin also mediates heterotypic interactions between epithelial cells and intraepithelial lymphocytes. A majority of sporadic colorectal adenomas and carcinomas contained somatic mutations in the APC gene, even in the smallest adenomas less than 1 cm in diameter. Similarities in the character and distribution of the APC mutations in both adenomas and carcinomas from sporadic and hereditary lesions strongly suggest that APC mutations are a very early event, if not an initiating event, in the development of most common colorectal tumours.

*Received 17 May 1996*
*Revised 15 August 1996*
*Accepted 20 August 1996*

*Correspondence to:* C Gespach, INSERM U55, Laboratoire Cancérogénèse et Différenciation de l'Epithélium Gastro-Intestinal, Hôpital Saint-Antoine, 75571 Paris 12, France

The stepwise accumulation of genetic alterations in colon cancer involves many oncogenic defects, including activation of the membrane-bound $pp60^{c\text{-}src}$ tyrosine kinase, also considered as an early event in the cancerous progression (Cartwright et al, 1994). Among the cellular targets of $pp60^{c\text{-}src}$ were identified the PI3 kinase, *ras*-GAP, PLCγ, PP2A, Shc, *raf*, *jun* and Fra2 (Courtneidge, 1994; Suzuki et al, 1994). Some of the oncogenic effects related to the activation of the $pp60^{c\text{-}src}$ kinase pathway are mimicked by the polyoma middle T antigen (Py-MT), the main transforming protein of polyomavirus. We demonstrated that Py-MT induced the tumorigenic conversion of the SV40 large T-immortalized rat intestinal cell line SLC-44 and enhanced the tumorigenicity of human colonic Caco-2 cells (Chastre et al, 1993). In polyomavirus (Py)-transformed cells, $pp60^{c\text{-}src}$ kinase activity in enhanced by the binding of Py-MT to $pp60^{c\text{-}src}$ (Courtneidge and Heber, 1987). Middle T antigen increases the tyrosine kinase activity of $pp60^{c\text{-}src}$ by preventing phosphorylation of Y527 (Courtneidge and Heber, 1987). Polyoma MT also binds the src family tyrosine kinases $pp62^{c\text{-}yes}$ and $pp62^{c\text{-}fyn}$, interacts with and stimulates the P13 kinase, PP2A, c-*raf*-1 and Shc (Courtneidge, 1994; Dilworth et al, 1994). Moreover, the Py-MT pathways activate phospholipase Cγ and members of the 14–3–3 protein family (Pallas et al, 1994). Py-MT therefore mimics some of the intermolecular interactions and activation of signalling pathways controlled by activated growth factor receptors.

Despite the identification of a number of genetic and molecular alterations involved in colorectal carcinogenesis, relatively little is known on their specific role and their functional cooperation to confer the malignant and invasive phenotypes. In this context, the purpose of this study was to determine the potential oncogenic role of the src and Py-MT signalling pathways in adenoma-to-carcinoma conversion. For this purpose, the premalignant adenoma cell line, PC AA/C1, derived from a FAP patient (Paraskeva et al, 1984) was transfected with a series of expression vectors encoding either the activated (Tyr527 → Phe527) form of $pp60^{src}$, the Py-MT oncogene, the Py early region alone or combined with normal c-*src*. We therefore characterized the tumorigenic potential and the pattern of differentiation and proliferation of these transfected PC AA/C1 cell derivatives. Since both APC and $pp60^{src}$ have been shown to be localized or associated with cell–cell adhesion effectors or cytoskeletal components, we investigated the invasive phenotype of the parental and transfected PC cell lines in relation to the expression of the receptor, tyrosine kinase Met, and its ligand hepatocyte growth factor / scatter factor (HGF/SF) as known effectors of invasion (Guan and Shalloway, 1992; Rubinfield et al, 1993; Rosen et al, 1994; Smith et al, 1994). We also analysed the expression and phosphorylation status of the E-cadherin–catenin cell adhesion complex binding the tyrosine kinase substrate $p120^{cas}$ (Daniel and Reynolds, 1995).

### MATERIALS AND METHODS

### Cell culture and human epithelial crypt preparation

The human colonic PC AA/C1 cell line (Paraskeva et al, 1984; Williams et al, 1990) was designated as the parental PC cell line throughout this study. Parental and transfected PC cells were routinely grown at 37°C on collagen-coated Petri dishes (Costar), in Dulbecco's modified Eagle medium (DMEM) containing 4.5 g $l^{-1}$ glucose, 20% fetal calf serum, 8 mM L-glutamine, antibiotics, 0.2 μU $ml^{-1}$ insulin and 1 μg $ml^{-1}$ hydrocortisone under a water-saturated atmosphere containing 5% carbon dioxide and 95% air. Cells were passaged weekly when reaching confluency in a 1:3 split ratio using trypsin/EDTA.

Specimens from patients who underwent surgery for FAP were obtained from the Centre de Chirurgie Digestive (Professor Parc, Hôpital Saint-Antoine, Paris, France). Human colonic epithelial crypts were obtained from fresh samples (Emami et al, 1989).

### Constructs and transfection

The retroviral vectors pLJ, kindly provided by Dr H Piwnica-Worms (Piwnica-Worms et al, 1987), contain the selection marker neomycine-resistance gene and the polyoma virus early region alone (pLJ-Py) or combined with either the normal (pLJ-C-Py) or mutated Tyr-527 → Phe-527 chicken src cDNA (pLJ-527-Py). We constructed the control vector pLJ-vect and the mutated (Tyr-527 → Phe-527) chicken src expression vector pLJ-msrc by excision of the Py early region from the pLJ-Py and pLJ-527-Py vectors using *Apa*I. The Py virus early region encodes the small, middle and large T oncoproteins. The control vector, Homer 6, and the corresponding pHO6MT1 vector recombined with the Py middle T oncogene were a generous gift from Dr D Spandidos (Chastre et al, 1993). Transfections were performed using the lipofection method (Lipofectin Reagent, Gibco BRL). The G418-resistant colonies were selected using 500 μg $ml^{-1}$ geneticin for 2 weeks (Sigma).

The GTP/GDP ratio on $p21^{ras}$ in the parental and transfected PC cell lines was measured according to the method of Burgering et al (1991), with minor modifications (Baron-Delage et al, 1994).

### Ultrastructural and histological analyses

Monolayers of PC cells were processed for electron microscopy, as previously described (Chastre et al, 1993). The histology of the tumours in nude mice and liver or lung was analysed after fixation in 4% formaldehyde, paraffin embedding and staining with haemoxylin phloxin safran or periodic acid–Schiff.

### Tumorigenicity in nude mice

Exponential cultures of PC cells and their derivatives were harvested using trypsin/EDTA and resuspended in phosphate-buffered saline (PBS). An inoculum of $10^7$ cells in 100 μl of PBS was then injected subcutaneously in the flank of female 4-week-old athymic nude mice. Tumour formation was assessed twice a month. The tumour volume was monitored by three-dimensional calliper rule measurements.

### In vitro invasion assays

Aggregates, cell suspensions or monolayer fragments from PC cells and their derivatives were confronted with precultured heart fragments from 9-day-old embryonic chick on top of semisolid agar medium. For evaluation of invasiveness, the interaction of the confronting human cells with the heart tissue was classified as grades 0 to IV, as previously described (Bracke et al, 1984).

As described by Vakaet et al (1991), 10-$cm^2$ wells were filled with 1.2 ml of a neutralized collagen G (type 1) solution (Seromed, Biochrom, Berlin, Germany) and incubated overnight at 37°C to allow gellification. Cells were harvested with trypsin/ EDTA and seeded on top of the collagen gels at a density of $10^5$ cells $ml^{-1}$ in 5 ml of culture medium. Cultures were then incubated at 37°C for 24–48 h and the depth of cell migration inside the gel was measured using an inverted microscope controlled by a computer-guided fine focus knob. Deep and superficial cells were counted in ten fields of 0.37 m $m^3$ (0.157 $mm^2$). The invasion index was expressed as the percentage of cells invaded into the gel over the total number of cells. An invasive index higher than 10% designated highly invasive cells (Vakaet et al, 1991; Vleminckx et al, 1991).

### RNA isolation and Northern blot analysis

Total RNA was isolated by guanidinium isothiocyanate extraction and caesium chloride density gradient ultracentrifugation. After denaturation, 20 μg of RNA was separated by electrophoresis

© Cancer Research Campaign 1997

through a 1% agarose – 2.2 M formaldehyde gel, transferred onto nylon membranes (Hybond N+, Amersham, UK) and UV-cross-linked (Stratalinker; Stratagene, CA, USA). The cDNA probes were: the chicken src cDNA corresponding to the 1.6-kb *BglI* fragment isolated from pLJ-C-Py; the Py-MT cDNA isolated from pHO6MT1 by *BamHI* (5.2-kb fragment); the 1.5-kb human elongation factor-1 cDNA isolated by *PstI* from the hEF1 plasmid was used as internal standard to assess the equality of the RNA loading.

### Expression of the HGF gene by RT–PCR and Southern blot

For RT–PCR analysis, RNA samples (2 μg) were reverse transcribed for 60 min at 37°C, using 200 U of Moloney murine leukaemia virus reverse transcriptase (Gibco BRL, France). The amplification consisted of 28–30 cycles (HGF) or 20–22 cycles (GAPDH) of denaturation for 30s at 94°C, annealing for 1 min at 58°C and a 2-min extension at 72°C in an automated thermal cycler (Techne, France). The reaction was initiated by a 5-min incubation at 94°C and ended after a 7-min extension at 72°C. PCR products were run on 1.5% agarose gels stained with ethidium bromide. For Southern analysis, PCR products were transferred to Hybond N+ membranes by alkali blotting and hybridized overnight with the internal probes end-labelled with [$\gamma^{32}$P]ATP. The amplification of the cDNA fragment extending from the K3 domain (exon VIII) to the 5′ portion of HGF b chain (exon XV) sequence (Miyazawa et al, 1991) was performed using the sense primer 5′ GGAATGGAATTCCATGTCAGCGTT-3′ (nucleotides 962–985) and antisense primer 5′-TCAAGTCTCGAGAAGGGAAACA-3′ (nucleotides 1603–1624). The expected size of the PCR product was 663 bp. The sequence of the corresponding internal probe was 5′-TGGGAACCAGATGCAAGTAAGCTG-3′ (nucleotides 1403–1425).

### Immunoprecipitation, kinase assay and Western blot

### pp60$^{src}$ and Py-MT

Methods for cell lysis, immunoprecipitation, kinase assays and Western blotting of the pp60$^{src}$ proteins have been described previously (Coutneidge and Heber, 1987). Cell lysates (100–200 μg of protein) were incubated for 60 min with either the monoclonal antibodies 327, which recognizes both human and chicken *src*, or EC10 specific for avian *src*, PAb α3C3 specific for the Py-MT antigen, or the rat anti-cst1 antiserum directed against the carboxy-terminal peptide shared by the *src* family kinases. For the immunoprecipitations, 20 μl of *S. aureus* (coated with rabbit anti-mouse IgGs for the MAbs) was then added for a further 30-min incubation. As a negative control, immunoprecipitations were performed using normal mouse IgG or rabbit serum.

Kinase assays were performed for 10 min at 30°C in 20 ml of 20 mM Hepes buffer (pH 7.2) containing 10 mM manganese chloride 1 μM ATP, 10 μCi [$\gamma^{32}$P]ATP (5000 Ci mmol$^{-1}$, Amersham) and 1.25 μg of heated and acid-denatured enolase. In order to evaluate the relative amount of src protein in immunoprecipitates, duplicate samples were analysed by Western blotting.

For the Western blots, total proteins or immunoprecipitates were submitted to electrophoresis in a 9% polyacrylamide gel and blotted to nitrocellulose membranes (Hybond-C extra, Amersham). Following two rinses in PBS for 5 min, the nitrocellulose was probed for 2 h with either the monoclonal antibodies MAb327 or PAb762 directed against the Py antigens and revealed by enhanced chemiluminescence Western detection system (ECL, Amersham).

### Met

Cell lysates were spun at 15 000 × ***g*** for 15 min, and the supernatants were immunoprecipitated after 2 h incubation with the MAb DO-24 directed against the extracellular domain of Met and 1 h incubation with protein A–Sepharose (Pharmacia, Uppsala, Sweden). The immunoprecipitates were then washed with the lysis buffer, denaturated by boiling in reducing sodium dodecyl sulphate (SDS) sample buffer and subjected to 7.5% SDS-polyacrylamide gel electrophoresis (SDS-PAGE).

In order to examine the tyrosine phosphorylation and the relative abundance of the Met receptor in parental and transfected PC cells, the same nitrocellulose-transferred proteins were first probed with the anti-phosphotyrosine antibodies MAb 05–321 (Upstate Biotechnology, NY, USA), stripped, and then incubated with the anti-Met MAb DL21. The phosphorylation level of Met is representative of its tyrosine kinase activity (Naldini et al, 1992). The relative abundance of the Met protein was also analysed on Western blots using whole-cell extracts and quantified by scanning densitometry of the radiographic films. For the HGF-induced activation of Met, the parental and transfected PC cells were starved of fetal calf serum for 17 h and then incubated in the presence or absence of HGF (500 U ml$^{-1}$).

### E-cadherin, α- and β-catenin, p120$^{cas}$ and APC

Whole-cell extracts were prepared in SDS sample buffer. Equal amounts of proteins were separated on a 7.5% SDS-PAGE gel and blotted to a PVDF membrane (Millipore, MA, USA). The following mouse IgG1 MAbs were used: Ab-1, directed to the N-terminal 29 amino acids of the APC protein (Oncogene Science, NY, USA); HECD-1, against E-cadherin (Takara Biomedicals, Otsu, Japan); anti-pp120, against p120$^{cas}$ (Transduction Laboratories, Kentucky, USA); PY20 anti P-Tyr (ICN, Ohio, USA), and two rabbit PAbs against α- and β-catenins specific peptides. Signals were visualized with anti-mouse IgG or anti-rabbit IgG alkaline phosphatase-conjugated IgG (Sigma, MO, USA) or with anti-mouse IgG biotinylated Ig (Amersham, UK), followed by streptavidin–horseradish peroxidase (HRP, Amersham). The HRP signal was visualized with the ECL system (Amersham).

For immunoprecipitation of E-cadherin and associated proteins, cells were labelled for 3 h with 125 μCi [$^{35}$S]methionine per 25-cm$^2$ flask and washed twice with PBS containing $Ca^{2+}$ and $Mg^{2+}$ and lysed with 300 μl of lysis buffer containing 0.5% Nonidet P-40, 10 mM Pefabloc SC (Merck, Germany), 0.5 μg ml$^{-1}$ leupeptin in PBS with $Ca^{2+}$ and $Mg^{2+}$. The lysates were cleared and adjusted to equal amounts of TCA-precipitable c.p.m. per volume. Up to 1 μg of HECD-1 Ab was used per aliquot for 3 h at 4°C. Immunoprecipitates were collected using protein G-Sepharose (Pharmacia) and separated on a 7.5% SDS gel.

Treatment of the PC cells with HGF (10 U ml$^{-1}$) was done for 24 h. Before lysis, cells were treated with 1 mM sodium vanadate and 2 mM hydrogen peroxide for 10 min. In each experiment, equal amounts of proteins were compared. E-cadherin and associated proteins in the lysates were immunoprecipitated with the MAbs HECD-1 or anti-pp 120. After centrifugation, the immunocomplexes were washed, boiled with SDS sample buffer, separated on a 7.5% SDS gel and blotted to a PVDF membrane. The blot was consecutively probed with mouse MAbs against phosphotyrosine (PY20), E-cadherin (HECD-1) or p120$^{cas}$ (anti-pp 120). This was

© Cancer Research Campaign 1997 

**Figure 1** Expression of chicken src, Py-MT/-LT and pp60$^{src}$ tyrosine kinase activity in parental and transfected human colonic PC cells. Parental PC cells (PC, lanes 1–2) were transfected either with the control vector PLJ-vect (PCvect, lanes 3–4), mutated chicken src (PCm-*src*, lanes 5–6), polyoma MT (PCMT, lanes 7–8), Py early region alone (PCPy, lanes 9–10) or combined with normal chicken src (PCPy/c-*src*, lanes 11–12). The even and uneven numbered lanes in **A**, **B** and **D** correspond to the immunoprecipitations using specific MAbs or normal mouse IgGs as control respectively. (**A**) Immunoprecipitation and Western blot of chicken src. Cell lysates (200 µg of protein) were immunoprecipitated with the MAb EC10 specific for avian src and resolved on a 7.5% SDS-PAGE. Chicken src was then revealed by Western blot, using the MAb 327 and the ECL detection system. The control immunoprecipitations performed with non-immune mouse antisera (uneven lanes) revealed an autoradiographic band of $M_r$ 50 000 that corresponds to the non-specific mouse IgGs. (**B**) Tyrosine kinase activity of chicken src. EC10 immunoprecipitates from 200 µg of protein were incubated with [$\gamma^{32}$P]ATP, with enolase as exogenous substrate and resolved on a 9% SDS-PAGE. Autoradiograph was performed for 8 h. (**C**) Western blot analysis of Py-MT and -LT. Cell lysates (100 µg of protein) were resolved on a 9% SDS-PAGE and analysed using the MAb PAb762, which recognized the Py-LT and -MT antigens. The viral oncoproteins were revealed by the ECL detection system. (**D**) Total src kinase activity. Ab327 immunoprecipitates obtained from 200 mg of protein lysates were incubated with [$\gamma^{32}$P]ATP and enolase. In view of the abundance of src in PCm-*src* cells, only 100 µg of protein was immunoprecipitated from this transfected cell line. Autoradiograph was performed for 8 h

**Figure 2** Association of the PyMT antigen with src kinase activity. Lysates from the cell lines PC (lanes 1 and 2), PCPy/csrc (lanes 3 and 4) or PCMT (lanes 5–8) were immunoprecipitated with either anti-src MAb 327 (lanes 2, 4 and 6) or anti-MT polyclonal serum α3C3 (lane 8) and incubated with [$\gamma^{32}$P]ATP for kinase assay. Control immunoprecipitations were performed using non-immune IgG (lanes 1, 3 and 5) or normal rabbit serum (lane 7). The migration positions of src and Py-MT phosphorylated *in vitro* are indicated. The arrow indicates an autoradiographic band with apparent $M_r$ 85 000 corresponding to a subunit of PI-3 kinase followed by biotinylated anti-mouse IgG and horseradish peroxidase-conjugated streptavidin. The signals were visualized with the ECL system.

### RESULTS

### Functional insertion of the transgenes

The human colonic adenomatous PC AA/C1 cell line (designated PC cells throughout this study) was subjected to lipofection in the presence of the control vector pLJvect, the pLJvect recombined with either mutated chicken src, the Py early region alone or combined with native chicken c-*src*. Transfections were also performed using the control vector, Homer 6, or the same vector recombined with the viral oncogene, Py-MT. The G418-resistant colonies were further selected by Northern blot according to the expression of the transfected oncogenes.

The functional insertion of chicken src cDNA in the PCm-*src* and PCPy/c-*src* cell lines was further investigated at the protein level by immunoprecipitation using the MAb EC10 specific for chicken *src*. Since this antibody is not efficient in Western blot, the chicken pp60$^{src}$ protein was first immunoprecipitated with EC 10 and then revealed by the MAb Ab327 (Figure 1A). The mutated or native chicken src proteins with an apparent $M_r$ of 60 000 were exclusively identified, respectively, in the transfected PCmsrc and PCPy/c-src cell lines (lanes 6 and 12).

In order to confirm the functional activity of the chicken *src* transgene in the PCm-*src* and PCPy/c-*src* cell lines, *src* kinase activity was assayed in the EC10 immunoprecipitates, using enolase as exogeneous substrate (Figure 1B). The phosphorylated enolase was identified as a major autoradiographic band with an apparent $M_r$ of 40 000 (lanes 6 and 12). In these samples, a minor band of $M_r$ 60 000 corresponding to autophosphorylated chicken src was also detected (data not shown).

The expression of the Py-MT and/or Py-LT oncogenes was assessed by Western blot analysis using the MAb PAb762. As shown in Figure 1C, the 55-kDa Py-MT antigen was detected in the PCMT, PCPy and PCPy/c-*src* cell lines (lanes 8, 10 and 12 respectively). As expected, an additional band with apparent $M_r$ 100 000 corresponding to Py-LT was observed in PC cells transfected by the Py early region (lanes 10 and 12).

To delineate the consequences of the insertions of the transgenes on the overall pp60$^{src}$ activity, human and chicken src were immunoprecipitated using the MAb327, followed by kinase assay (Figure 1D). Total *src* tyrosine kinase activity was markedly increased in the PCm-*src* and PCPy/c-*src* cell lines transfected by wild-type or mutated chicken src (lanes 6 and 12), compared with parental and control PCvect cells (lanes 2 and 4) or epithelial crypts isolated from normal human colon mucosa (data not shown). In contrast, src activity was not enhanced in PC cells transfected by Py-MT (lane 8) or the Py early region (lane 10). This observation is not related to a preferential interaction of the Py-MT antigen with the human pp62$^{c\text{-}yes}$ or pp59$^{c\text{-}fyn}$ tyrosine kinases, as a kinase assay using the antibody αcst1, which recognizes *src*, *yes* and *fyn*, did not reveal any increase in tyrosine kinase activity in PC cells transfected by the Py early region or MT alone (data not shown). The interaction between Py-MT antigen and src was therefore investigated in the transfected PCMT and PCPy/c-*src* cells after immunoprecipitation kinase assay (Figure 2), using the *src* MAb 327 (lanes 1–6) and the Py-MT PAb α3C3 (lanes 7–8). The Py-MT antigen ($M_r$ 55 000) was found to be phosphorylated and associated with pp60$^{src}$ in *src* immunoprecipitates

 © Cancer Research Campaign 1997

**Figure 3** Induction of the tumorigenic potential in parental human colonic PC cells after oncogene insertion. Nude mice were inoculated subcutaneously with $10^7$ cells, before (PC) or after transfection by mutated chicken src (PCmsrc), Py-MT (PCMT), the Py early region alone (PCPy) or combined with normal chicken src (PCPy/c-src). Each point is the mean of 5–10 determinations representative of 2–4 separate experiments prepared from PCMT cells (lane 6) and PCPy/c-*src* cells (lane 4). High levels of the autophosphorylated form of *src* ($M_r$ 60 000) were observed in PCPy/c-*src* cells. This strong autophosphorylation signal reflects the increased total pp60$^{c\text{-}src}$ activity previously observed in this cell line (Figure 1D), compared with PC and PCMT cells. The kinase assay performed on Py-MT immunoprecipitates prepared from PCMT cells (lane 8) allowed the identification of an autoradiographic band, corresponding to the phosphorylated Py-MT antigen.

Our data suggest that the viral antigen interacts with the src tyrosine kinase, leading to the phosphorylation of Py-MT and formation of molecular associations between the *src*/Py-MT complex and different targets of Py-MT, such as the 85-kDa subunit of the Pl-3 kinase, as shown in Figure 2 (lanes 4, 6 and 8).

### Status of p21$^{ras}$, cell proliferation and differentiation

As the p21$^{ras}$ protein is another downstream effector of *src* and Py-MT, we analysed the GDP/GTP ratio on *ras* in the parental PC cells and their derivatives. We observed that PC cells exhibited a high proportion of GTP bound to p21$^{ras}$ (49%), because this cell line harbours a substitution of the Gly-12 residue for a valine in one allele of the Ki-*ras* gene (Farr et al, 1988; Chastre et al, 1993). This constitutive activation of mutated Ki-*ras* in PC cells was not further increased after the insertion of the oncogenes (data not shown), and compares with the proportion of GTP bound to p21$^{ras}$ (45–48%) observed in Caco-2 cells transfected by oncogenic *ras* (Baron-Delage et al, 1994). The doubling time of the transfected PC cell lines (40 h) was similar to that observed in the parental cells: 38–40 h for the PCm-*src*, PCPy and PCPy/c-*src* cell lines, or slightly higher in PCMT cells (47 h).

The oncogene-transfected PC cells did not show striking morphological changes by phase-contrast microscopy. Detailed differences shown by exponentially growing PCm-*src* cells consisted in the appearance of pseudopod-like structures and ruffling at the cellular membrane, suggesting decreased cell–cell or cell–matrix adhesion properties (data not shown). Transmission electron microscopy showed that the transformation of the highly differentiated PC cell line by src, Py-MT alone or combined with PyLT and small T did not result in loss of epithelial organization or morphological phenotype contrary to our previous observations on ras- and Py-MT- transformed Caco-2 enterocytes (Chastre et al, 1993; Baron-Delage, 1996). Oncogene-transfected PC cells exhibited typical apical tight junctions and retention of the epithelial polarization. Thus, our results raise the hypothesis that the FAP-derived colonic PC cell line and mucinous differentiation exert a dominant control on the oncogenic functions mediated by src, Py-MT and -LT regarding cell proliferation and differentiation.

### Introduction of src and polyoma virus oncogenes induce tumorigenicity in nude mice

Following the introduction of $10^7$ cells into nude mice, the parental PC cell line did not produce any tumour 1 year after the injections (Figure 3). The same observation was made for the control vector cells, PCvect or PCH (data not shown). In contrast, PCMT cells grew very slowly to produce significant tumour formation by

**Table 1** Effect of the oncogenes src, polyoma middle T and large T on the invasive potential of the PC cell lines

| | Invasion assays | | | | | | |
|---|---|---|---|---|---|---|---|
| | Type I collagen | | | | Chick heart assay | | |
| | –HGF | | +HGF | | | | |
| Cell lines | Index (%)[a] | Depth (μm)[b] | Index(%)[a] | Depth (μm)[b] | Adherent(*n*/*n*)[c] | Invasion(*n*/*n*)[d] | Grade[e] |
| PC | 0.1±0.1 | 25 | 0.0±0.0 | 0 | 11/27 | 2/11 | I II III |
| PCm-*src* | 0.5±0.3 | 25 | 5.9±2.1[f] | 50 | 20/29 | 3/20 | I II III |
| PCMT | 0.0±0.0 | 0 | 0.2 ±0.2 | 50 | 10/11 | 0/10 | I |
| PCPy/c-*src* | 0.0±0.0 | 0 | 0.0±0.0 | 0 | 36/40 | 3/36 | I II III |
| MCF-7 | 0.0±0.0 | 0 | | | | | |
| DHB-FIB | 13.6±2.8 | 100 | | | | | |

[a]Percentage of invasive cells after 24 h of incubation. [b]Maximum depth of invasion in collagen gel. [c]Number of cultures showing adherence of confronting cells to the heart fragment over total number of cultures. [d]Number of cultures showing invasion over total number examined. [e]Invasion was scored as follows: I, cancer cells were separated from the cardiac muscle by fibroblastic cells; II, cancer cells were apposed to the cardiac muscle (non-invasive); III, occupation of less than half the heart tissue by cancer cells (invasive). [f]Significantly different at $P$<0.005 from untreated cells.

**Figure 4** Immunoprecipitation and Western blot analysis of Met in parental and transfected human colonic epithelial PC cells. Cell lysates (1 mg of protein) were immunoprecipitated with the MAb DO-24 and resolved on a 7.5% SDS-PAGE. The Met protein was then revealed by the MAb DL-021 against the C-terminal tail of the HGF receptor and the ECL detection system. Hybridization revealed the mature Met receptor β chain (p145) and the uncleaved αβ Met precursor (p170)

**Figure 5** Expression and phosphorylation levels of E-cadherin and associated proteins in parental and transfected PC cells. (**A**) Cells were labelled with [$^{35}$S]methionine, and lysates were incubated with MAb HECD-1 against E-cadherin. The protein complexes were resolved on a 7.5% SDS polyacrylamide gel. (**B**) Cell lysates were immunoprecipitated with the MAb HECD-1, resolved on a 7.5% SDS polyacrylamide gel and blotted to a PVDF membrane. The tyrosine-phosphorylated proteins of the cadherin complex were revealed by the MAb PY20 and the ECL system. Control immunoprecipitation was performed without using HECD-1. Migration position of E-cadherin is indicated by the arrow

8 months. Thereafter, the PCMT tumours grew at a rapid and relatively uniform rate and reached 1 cm in diameter 1 year after the injections. Tumour incidence of PCMT cells was 77%. In contrast, PC cells transfected by the Py early region alone or combined with c-src formed rapidly progressing tumours characterized by a shorter latency time (2–3 months) in 72–90% of animals. An intermediate situation was observed for the PCmsrc tumours (latency 4 months) observed in 82% of mice.

Histopathological analysis of the xenografts indicated that the oncogene-transfected PC cells produced moderately to well-differentiated adenocarcinomas. The dysplastic material, classified as human glandular tumours, contained areas of mucosecretion in luminar spaces, as evidenced by periodic acid–Schiff and alcian blue positivity (data not shown). The PCm-*src* cells produced highly differentiated tumours characterized by an elevated secretion of mucins.

### Introduction of mutated src confer the HGF-dependent invasiveness in collagen gels

No metastases were histologically evidenced in peritoneal cavity, liver or lungs in nude mice injected subcutaneously with the more aggressive PCPy/c-*src* cells, as well as the other oncogene-transfected cells. The invasiveness of PC cells and their derivatives was further assessed in invasion assays in vitro that scored cell penetration into three-dimensional collagen matrices and invasion and destruction of embryonic chick heart fragments (Vakaert et al, 1991). The parental and transfected PC cell lines failed to invade into the collagen gel, like MCF-7/AZ cells, which were used as negative control (Table 1). The positive control rat myofibroblastic cells DHD-FIB were invasive in this assay.

Since the paracrine factor HGF secreted by stromal cells acts on the proliferation and the scattering of epithelial cells (Rosen et al, 1994), we investigated the invasive properties of the parental PC cells and their derivatives in the presence of HGF/SF. As shown in Table 1, PCmsrc cells became invasive into collagen gels after HGF treatment. In contrast, PCMT and PCPy/c-*src* cells remained non-invasive. These results indicate that HGF/SF alone is not sufficient to confer the invasive phenotype to PC cells, but that it cooperates with the activated pp60$^{src}$ transduction pathway to induce invasion in PCmsrc. Nevertheless, the absence of response of the other cell lines to HGF is not related to a dysfunction of the HGF receptor Met, since the treatment of the parental and oncogene-transfected PC cells is associated with tyrosine phosphorylation of the Met receptor, corresponding to its activation (data not shown). The parental PC cells and their derivatives were also tested for their invasiveness using embryonic chick heart fragments (Table 1). Invasion occurred in only a few cultures and was limited to the presence of a few cells inside the chick heart. Addition of HGF in this assay had no effect on the invasion properties of PC cell lines (data not shown).

### Met and HGF expression in parental and transfected PC cells

In order to explain the differential responses to HGF observed in the collagen invasion assay for the PCmsrc and PCPy/c-*src* cells, we analysed the relative expression of the HGF receptor Met. A significant four-fold increase ($P<0.01$, $n=8$ experiments) of the Met protein accumulation was observed in PCm-*src* cells and the other transfected PC cells (2- to 2.6-fold). Figure 4 is a representative

 © Cancer Research Campaign 1997

**Figure 6** Effect of HGF on accumulation and phosphorylation levels of E-cadherin and p120*cas* in PC and PCm-*src* cells. Lysates from cells treated or untreated by 10 U ml$^{-1}$ HGF for 24 h were immunoprecipitated with the antibodies HECD-1 (directed to E-cadherin) or anti-pp 120, resolved on a 7.5% SDS polyacrylamide gel and blotted to a PVDF membrane. (**A**) The E-cadherin protein and the p120*cas* isoforms were detected by HECD-1 and anti-pp120 respectively. (**B**) The tyrosine phosphorylated proteins of the cadherin and p120*cas* complex were revealed by the MAb PY20 and the ECL system. Control immunoprecipitations in PCm-src (negative control) were performed in the absence of HECD-1 or anti-p120 antibodies

Western blot of the p145 and p170 forms of the HGF receptor identified after immunoprecipitation. Similar results were obtained by direct Western blot. The overexpression of Met may result from transcriptional or translational deregulation owing to the activation of the src and Py-MT transduction pathways. However, another possibility arises from two recent studies suggesting that: (1) the HGF response is autoamplified by inducing the MET receptor gene (Boccaccio et al, 1994); (2) autocrine activation of the Met receptor was observed in human osteosarcoma and lung carcinoma cells secreting HGF (Tsao et al, 1993). Consequently, we analysed by RT–PCR analysis and Southern blotting whether the enhanced expression of the Met oncoprotein in oncogene-transfected PC cell lines is related to endogenous HGF. In parental and oncogene-transfected PC cells, clear signals for HGF transcript were seen (PCR product = 663 bp) as well as in Caco-2 and HT-29 human colonic adenocarcinoma cells and colonic crypts isolated from a FAP patient (data not shown). Strong signals were also observed in human colonic mucosa.

### Expression of APC, E-cadherin and associated proteins

The FAP-derived PC cell line and derivatives, PCmsrc and PCPy/c-src, were analysed for APC protein expression. In agreement with the literature, a truncated APC protein was detected in the PC cell line. There were no apparent differences in APC expression among the three PC cell lines (data not shown).

Upon immunoprecipitation with an E-cadherin-specific MAb, all PC cells lines analysed expressed the same molecular complex (Figure 5A). In association with E-cadherin, one finds α-catenin, β-catenin and other proteins that might comprise plakoglobulin (γ-catenin) and the p120*cas* isoforms (catenin-related src substrates). Taking into account the variable yields of the immunoprecipitates (as apparent from the non-specific 75-kDa band) and several Western blot experiments using antibodies against E-cadherin or any of the catenins mentioned (data not shown), one may conclude that neither large quantitative nor striking qualitative differences exist for the E-cadherin–catenin complexes in the various PC cell lines. Regarding p120*cas*, several isoforms were detected in the PC cells, but they were all of the smaller epithelial type (< 120 kDa; Reynolds et al, 1994). Most of them were found to be poorly associated with the E-cadherin–catenin complex (see also Figure 6A).

### Effect of src and HGF on tyrosine-specific phosphorylation of E-cadherin, p120*cas* and associated proteins

Parental PC cells were compared to derivative cell lines for tyrosine-specific (Tyr) phosphorylation of molecular elements of the E-cadherin–catenin complexes. As expected, such phosphorylation was particularly evident upon src transformation, with mutated *src* showing more activity in PCm-*src* cells than overexpressed normal src in PCPy/c-*src* cells (Figure 5B). Besides strong tyrosine phosphorylation of a 120-kDa band, phosphorylated 97- and 85-kDa bands were obvious too (Figure 6B). Reprobing the blot with appropriate antibodies indicated comigration of the 120-kDa band with E-cadherin, of the 97-kDa band with one of the 120*cas* isoforms, and of the 85-kDa band with β-catenin.

In the next experiment; the effect of HGF on Tyr phosphorylation of E-cadherin and catenins in parental PC *vs* PCm-*src* cells was assessed. As shown in Figure 6B, 24 h treatment with HGF did enhance slightly the Tyr phosphorylation of the tentative E-cadherin band in PCm-*src* cells (arrowed 120-kDa band), whereas the lower bands were either dephosphorylated or dissociated from the E-cadherin complex. The observed increase of phosphorylation was not caused by increased protein level, as the same blot showed slightly less E-cadherin upon probing with an E-cadherin-specific antibody (Figure 6A). Immunoprecipitation with MAb anti-pp120*cas* showed no 120-kDa band when blotted with the same antibodies (Figure 6A), whereas blotting with anti-phosphotyrosine antibodies yielded a 120-kDa band in the PCmsrc lane (Figure 6B). It is likely that this band represents E-cadherin coprecipitating with p120*cas* (Reynolds et al, 1994) and phosphorylated on Tyr residues by src activity. Tyrosine phosphorylation of the p120*cas* isoforms was already observed in parental PC cells and apparently was not much influenced by additional src activity in PCm-*src* cells.

### DISCUSSION

We have demonstrated that the src and Py-MT oncogenes induced the adenoma-to-carcinoma progression in colonic epithelial cells derived from a FAP patient, but failed to induce the formation of spontaneous metastases and invasiveness in vitro.

Genetic alterations representative of the early stages of human colorectal carcinogenesis, including truncating mutant APC and activated Ki-*ras* genes, were previously identified in the recipient adenomatous cell line and were confirmed in the present study (Farr et al, 1988; Smith et al, 1993). These alterations already occurred in early stages, e.g. dysplastic adenomas and colitis-associated neoplasia (Redson et al, 1995). The inherited APC inactivation combined with oncogenic *ras* in PC cells did not induce tumorigenicity in the nude mice. This progression was accomplished upon introduction of either Py-MT, mutated *src*, the Py early region alone or combined with c-*src*. Since p21$^{ras}$, one of the downstream effectors of *src*, is not further activated in oncogene-transfected PC cells, one can postulate a crucial role for *ras*-independent pathways in the *src*- and Py-MT-induced neoplastic progression. In this context, the contribution of several oncogenic events has been hypothesized to be required for the formation of a malignant tumour, and these arise from benign tumours, which also require genetic mutations. The adenoma-to-carcinoma conversion is associated with Met overexpression in all the oncogene-transfected PC cells. Recent observations indicate that transfection of MET in NIH 3T3 fibroblasts promotes colony formation in soft agar in the presence of HGF (Giordano et al, 1993). Our results, therefore, provide a new hypothesis related to a connection between activation of the *src*/Py-MT oncogenic pathways and the frequent overexpression of Met observed in human gastrointestinal tumours (Di Renzo et al, 1995). As HGF was recently shown to exert autoinduction on the transcription of MET, one might predict that *src* and PyMT exert similar actions via the response elements, AP1 and ets1, or other regulatory sequences identified in the MET-promoter region (Gambarotta et al, 1994). Py-MT alone induced low tumorigenic potential in PCMT cells with long latency periods, compared with PCm-*src* cells. Consistent with our interpretation that the src pathway is not fully activated by Py-MT in human colonic epithelial cells, only a small fraction of the viral middle T antigen was previously shown to be associated with an equally small fraction of pp60$^{src}$ (Courtneidge and Heber, 1987). Moreover, the induction of visceral haemangiomas and cell transformation by Py-MT has been reported in *src*-null mice (Thomas et al, 1993). Further studies should identify the src-independent pathways involved in the Py-MT-mediated neoplastic progression of the human colonic epithelial cell lines, Caco-2 and HT-29 (Chastre et al, 1993), and PC/AA/C1 in the present study. The most agressive tumours produced by PCPy or PCPy/c-*src* cells compare well with the malignant properties of the human colonic Caco-2 cells, which display a low tumorigenic potential in the nude mice (Chastre et al, 1993).

The invasion potential of the PCm-*src* cells observed here in collagen gels was revealed after the addition of HGF and correlates with a dramatic increase in pp60$^{src}$ tyrosine kinase activity. The levels of the *src* kinase may therefore play a key role in further progression. Increased expression and activity of c-src has been correlated with human metastases when fresh specimens were analysed (Talamonti et al, 1993), suggesting that src might be involved at both early and late stages of the neoplastic progression of human colonic epithelia. No studies have yet addressed a potential role for wild type *src* alone. Such a PC-transfected cell line would provide additional information with respect to the degree of src expression and activity required for tumorigenicity and invasiveness. Indeed, the adenoma–carcinoma transition is induced in the PC cell line by a mutated c-*src*, although *src* activation in human colon cancer is related to upstream molecular mechanisms and is not the result of mutation. Thus, HGF may further increase pp60$^{src}$ activity or activate another HGF-dependent pathway. In this connection, this report is the first description of a possible co-operation between activated src and Met receptor/ HGF pathways in cell invasion. Invasiveness was not induced by HGF in the PCMT and PCPy/c-*src* cells, suggesting that Py-MT is not sufficient to confer the HGF-dependent invasive potential. The presence of phosphorylated Pl3- kinase in the Py-MT and src immunoprecipitates from PCMT and PCPy/c-*src* cells also indicates that the other effector systems of Py-MT, including the Pl3 kinase, do not cooperate with the HGF/Met pathways to induce the invasive phenotype in Py-MT-transfected cells. Oncogenic *src* was previously shown to modulate many effector systems involved in cell invasion and motility, namely cell–cell contacts, cell–matrix interactions and proteases, e.g. transin, cathepsin and urokinase plasminogen activator (Gal and Gottesman, 1986; Bell et al, 1993). For example, v-*src* regulates the expression of integrins and extracellular matrix components, which play a major role in Met receptor signalling and HGF bioavailability (Plantefaber and Hynes, 1989; Santos et al, 1993). These mechanisms might be involved in the cascade of regulations that link src and the HGF-dependent invasiveness of PCm-*src* cells. Activation of src or Met promotes invasiveness through the destabilization of intercellular junctions, e.g. integrins–FAK and cadherins–catenin adhesion complexes (Guan and Shalloway, 1992).

We found no evidence for loss of cadherin function, regarding cell–cell adhesion and retention of epithelial polarization and formation of apical tight junctions in oncogene-transfected PC cells. E-cadherin-mediated cell adhesion is negatively regulated by phosphorylation of catenins or via other proteins that bind the cytoplasmic domain of cadherin molecules and link them to the cytoskeleton (Ozawa et al, 1989; Rubinfield et al, 1993). The E-cadherins–catenin complex proteins are major constituents of the adherens junctions that are regulated by mitogenic and oncogenic signals. We have shown here that activated src triggers prominent Tyr phosphorylation of a 120-kDa protein, tentatively E-cadherin, which was further enhanced after HGF treatment. In contrast, tyrosine phosphorylation of either p120$^{cas}$ isoforms or catenins does not seem to play a major role in src- and HGF-induced progression in PC cell lines. Our data on PCm-*src* cells suggest that E-cadherin serves as both *src* and Met substrate. It is therefore tempting to propose the HGF-increased Tyr phosphorylation of E-cadherin as causally related to the HGF-induced invasiveness of PCm-*src* cells into collagen gels. Taken together, this may indicate a convergence between activated *src* and Met pathways at the level of cadherin phosphorylation or intracellular mechanisms regulating cell invasion and mobility. Another interesting finding concerns the identification of HGF transcripts in the adenomatous PC cells and their derivatives as well as in Caco-2 and HT-29 cell lines. Our results raised the potential implication of autocrine and paracrine loops between HGF and Met during the progression of human colorectal cancers. However, the invasive potential of PCm-*src* in collagen gels was apparent only after the addition of biologically active HGF, suggesting that endogenous HGF was synthesized at low levels or remained as immature pro-HGF.

© Cancer Research Campaign 1997

In conclusion, we have shown that the src, Py-MT and -LT oncogenes induced different grades of tumorigenicity in the human colonic PC/AA/C1 cell line. Sustained activation of the src and HGF/Met receptor pathways in transfected PCm-*src* cells induced invasiveness. A potential role of the src and HGF/ Met oncogenes at early and late stages of the cancerous transformation is therefore proposed. To our knowledge, the HGF-dependent invasion by PCm-*src* cells is the first example of human colonic epithelial cells capable of invading collagen gels. This model should help (1) to identify differentially expressed messages at crucial transitions, including genes which are transcriptionally induced or suppressed by src and HGF/Met; (2) to study pharmacological agents, molecular or genetic elements controlling the growth and invasion of the oncogene-transfected PC cells.

### ACKNOWLEDGEMENTS

We gratefully acknowledge Dr Piwnica-Worms (Harvard Medical School, Beth Israel Hospital, Boston, USA) for providing the expression vectors pLJ, pLJ(C) and pLJ(527), Dr S Dilworth (RPMS, Hammersmith Hospital, London, UK) for the generous gift of the PAb 762. This work was supported by research grants and doctoral fellowships (S E and S D) from the Association de la Recherche Contre le Cancer (ARC) and la Ligue Nationale Contre le Cancer.

### REFERENCES

Baron-Delage S, Capeau J, Barbu V, Chastre E, Levy P, Gespach C and Cherqui G (1994) Reduced insulin receptor expression and function in human colonic Caco-2 cells by ras and polyoma middle T oncogenes. *J Biol Chem* **269**: 18686–18693

Baron-Delage S, Mahraoui L, Cadoret A, Veissiere D, Taillemite JL, Chastre E, Gespach C, Zweibaum A, Capeau J, Brot-Laroche E and Cherqui G (1996) Deregulation of hexose transporter expression in Caco-2 cells by ras and polyoma middle T oncogenes. *Am J Physiol* **270**: G314–G323

Boccaccio C, Gaudino G, Gambarotta G, Galimi F and Comoglio PM (1994) Hepatocyte growth factor (HGF) receptor expression is inducible and is part of the delayed-early response to HGF. *J Biol Chem* **269**: 12846–12851

Bracke ME, van Cauwenberge R and Mareel M (1984) (+)- Catechin inhibits the invasion of malignant fibrosarcoma cells into chick heart *in vitro*. *Clin Exp Metast* **2**: 161–170

Bell SM, Connolly DC, Maihle NJ and Degen JL (1993) Differential modulation of plasminogen activator by oncogene-encoded protein kinases. *Mol Cell Biol* **13**: 5888–5897

Cartwright CA, Coad CA and Egbert BM (1994) $pp60^{c\text{-}src}$ activation in human colon carcinoma. *J Clin Invest* **93**: 509–515

Chastre E, Empereur S, Di Gioia Y, El Mahdani N, Mareel M, Vleminckx K, Van Roy F, Bex V, Emami S, Splandidos DA and Gespach C (1993) Neoplastic progression of human and rat intestinal cell lines after transfer of the ras and polyoma middle T oncogenes. *Gastroenterology* **105**: 1776–1789

Courtneidge SA and Heber A (1987) An 81 kd protein complexed with middle T antigen and $pp60^{c\text{-}src}$: a possible phosphatidylinositol kinase. *Cell* **50**: 1031–1037

Courtneidge SA (1994) Protein tyrosine kinases, with emphasis on the src family. *Semin Cancer Biol* **5**: 239–246

Daniel J and Reynolds A (1995) The tyrosine kinase substrate $p120^{cas}$ bind directly to E-cadherin but not the adenomatous polyposis Coli protein or α-catenin. *Mol Cell Biol* **15**: 4819–4824

Dilworth SM, Brewster CEP, Jones MD, Lanfrancone L, Pelicci G and Pelicci PG (1994) Transformation by polyoma virus middle T-antigen involves the binding and tyrosine phosphorylation of *Shc*. *Nature* **367**: 87–90

Di Renzo MF, Olivero M, Giacomini A, Porte H, Chastre E, Mirossay L, Nordlinger B, Bretti S, Bottardi S, Giordano S, Plebani M, Gespach C and Comoglio PM (1995) Overexpression and amplification of the Met/HGF receptor gene during the progression of colorectal cancer. *Clin Cancer Res* **1**: 147–154

Emami S, Mir L, Gespach C and Rosselin G (1989) Transfection of rat intestinal epithelial cells by viral oncogenes: establishment and characterization of the E1A-immortalized SLC-11 cell line. *Proc Natl Acad Sci USA* **86**: 3194–3198

Farr CJ, Marshall CJ, Easty DJ, Wright NA, Powell SC and Paraskeva C (1988) A study of ras mutations in colonic adenomas from familial polyposis coli patients. *Oncogene* **3**: 673–678

Fearon ER and Vogelstein B (1990) A genetic model for colorectal tumorigenesis. *Cell* **61**: 759–767

Frixen UH, Behrens J, Sachs M, Eberle G, Voss B, Warda A, Löchner D and Birchmeier W (1991) E-cadherin-mediated cell–cell adhesion prevents invasiveness of human carcinomas cells. *J Cell Biol* **113**: 173–185

Gal S and Gottesman MM (1986) The major excreted protein of transformed fibroblasts is an activable acid-protease. *J Biol Chem* **5**: 1760–1765

Gambarotta G, Pistoi S, Giordano S, Comoglio PM and Santoro C (1994) Structure and inducible regulation of the human MET promoter. *J Biol Chem* **269**: 12852–12857

Giordano S, Zhen Z, Medico E, Gaudino G, Galimi F and Comoglio PM (1993) Transfer of motogenic and invasive response to scatter factor hepatocyte growth factor by transfection of human Met protooncogene. *Proc Natl Acad Sci USA* **90**: 649–653

Groden J, Thliveris A, Samowitz W, Carlson M, Gelbert L, Albertsen H, Joslyn G, Stevens J, Spirio L, Robertson M, Sargeant L, Krapcho K, Wolff E, Burt R, Hughes JP, Warrington J, Melherson J, Warmuth J, Le Paslier D, Abderrahim H, Cohen D, Leppert M and White R (1991) Identification and characterization of the familial adenomatous polyposis gene. *Cell* **66**: 589–600

Guan J-L and Shalloway D (1992) Regulation of focal adhesion-associated protein kinase by both cellular adhesion and oncogenic transformation. *Nature* **358**: 690–692

Hülsken J, Birchmeier W and Behrens J (1994) E-cadherin and APC compete for the interaction with β-catenin and the cytoskeleton. *J Cell Biol* **127**: 2061–2069

Kinzler KW, Nilbert MC, Su LK, Vogelstein B, Bryan TM, Levy DB, Smith KJ, Preisinger AC, Hedge P, McKechnie D, Finnear R, Markham A, Groffen J, Powell SM, Zilz N, Beazer-Barclay, Y, Bryan TM, Hamilton SR, Thibodeau SN, Vogelstein B and Kinzler KW (1992) Identification of FAP locus genes from chromosome 5q21. *Nature* **359**: 235–237

Miyazawa K, Kitamura A and Kitamura N (1991) Structural organization and the transcription initiation site of the human hepatocyte growth factor gene. *Biochemistry* **30**: 9170–9176

Naldini L, Tamagnone L, Vigna E, Sachs M, Hartmann G, Birchmeier W, Daikuhura Y, Tsubouchi H, Blasi F and Comoglio PM (1992) Extracellular proteolytic cleavage by urokinase is required for activation of hepatocyte growth factor/scatter factor. *EMBO J* **11**: 4825–4833

Ozawa M, Baribault H and Kemler R (1989) The cytoplasmic domain of the cell adhesion molecule ovomorulin associates with three independent proteins structurally related in different species. *EMBO J* **8**: 1711–1717

Pallas DC, Fu H, Haehnel LC, Weller W, Collier RJ and Roberts TM (1994) Association of polyomavirus middle tumor antigen with 14–3–3 proteins. *Science* **265**: 535–537

Paraskeva C, Buckle BG, Sheer D and Wigley CB (1984) The isolation and characterization of colorectal epithelial cell lines at different stages in malignant transformation from familial polyposis coli patients. *Int J Cancer* **34**: 49–56

Piwnica-Worms H, Saunders KB, Roberts TM, Smith AE and Cheng SH (1987) Tyrosine phosphorylation regulates the biochemical and biological activity of $pp60^{src}$. *Cell* **49**: 75–82

Plantefaber LC and Hynes RO (1989) Change in integrin receptors on oncogenically transformed cells. *Cell* **56**: 281–290

Redston MS, Papadopoulos N, Caldas C, Kinzler KW and Kern SE (1995) Common occurrence of APC and K-*Ras* gene mutations in the spectrum of colitis-associated neoplasias. *Gastroenterology* **108**: 383–392

Reynolds AB, Daniel J, McCrea PD, Wheelock MJ, Wu J and Zhang Z (1994) Identification of a new catenin: the tyrosine substrate $p120^{cas}$ associate with E-cadherin complexes. *Mol Cell Biol* **14**: 8333–8342

Rosen EM, Nigam SK and Goldberg ID (1994) Scatter factor and the c-Met receptor: a paradigm for mesenchymal/epithelial interaction. *J Cell Biol* **127**: 1783–1787

Rubinfeld B, Souza B, Albert I, Müller O, Chamberlain SH, Masiarz FR, Munemitsu S and Polakis P (1993) Association of the APC gene product with β-catenin. *Science* **262**: 1731–1734

Santos OFP, Moira LA, Rosen EM and Nigam SK (1993) Involvement of hepatocyte growth factor in kidney development. *Dev Biol* **159**: 538–545

© Cancer Research Campaign 1997

Smith KJ, Johnson KA, Bryan TM, Hill DE, Markowitz S, Willson JKV, Paraskeva C, Petersen GM, Hamilton SR, Vogelstein B and Kinzler KW (1993) The APC gene product in normal and tumor cells. *Proc Natl Acad Sci USA* **90**: 2846–2850

Smith KJ, Levy DB, Maupin P, Pollard TD, Vogelstein B and Kinzler KW (1994) Wild-type but not mutant APC associates with microtubule cytoskeletal. *Cancer Res* **54**: 3672–3675

Su LK, Vogelstein B and Kinzler KW (1993) Association of the APC tumor suppressor protein with catenins. *Science* **262**: 1734–1737

Suzuki T, Murakami M, Onai N, Fukuda E, Hashimoto Y, Sonobe MH, Kameda T, Ichinose M, Miki K and Iba H (1994) Analysis of AP-1 function in cellular transformation pathways. *J Virol* **68**: 3527–3535

Talamonti, M, Roh M, Curley S and Gallick G; (1993) Increase in activity and level of $pp60^{c\text{-}src}$ in progressive stages of human colorectal cancer. *J Clin Invest* **91**: 53–60

Thomas JE, Aguzzi A, Soriano P, Wagner EF and Brugge JS (1993) Induction of tumor formation and cell transformation by polyoma middle T antigen in the absence of src. *Oncogene* **8**: 2521–2529

Tsao MS, Zhu H, Giaid A, Viallet J, Nakamura T and Park M (1993) Hepatocyte growth factor/scatter factor is an autocrine factor for human bronchial epithelia and lung carcinoma cells. *Cell Growth Different* **4**: 571–579

Vakaet L Jr, Vleminckx K, Van Roy F and Mareel M (1991) Numerical evaluation of the invasion of closely related cell lines into collagen type I gels. *Invasion Metast* **11**: 249–260

Vleminckx K, Vakaet L Jr Mareel M, Fiers W and Van Roy F (1991) Genetic manipulation of E-cadherin expression by epithelial tumor cells reveals an invasion suppressor role. *Cell* **66**: 107–119

Williams AC, Harper SJ and Paraskeva C (1990) Neoplastic transformation of a human colonic cell line: *in vitro* evidence for the adenoma to carcinoma sequence. *Cancer Res* **50**: 4724–4730

*© Cancer Research Campaign 1997*